# Supplementary material for: Coding and non-coding roles of MOCCI (C15ORF48) coordinate to regulate host inflammation and immunity
Source: Nat Commun. 2021 Apr 9;12:2130. doi: 10.1038/s41467-021-22397-5 (PMC8035321; doi:10.1038/s41467-021-22397-5)
Supplement: Supplementary file 1 — Supplementary Information [file 41467_2021_22397_MOESM1_ESM.pdf]

Supplementary Information for

Coding and non-coding roles of MOCCI (C15ORF48) coordinate to regulate host inflammation and immunity

Cheryl Q.E. Lee[1], Baptiste Kerouanton[2], Sonia Chothani[2], Shan Zhang[2], Ying Chen[3], Chinmay Kumar Mantri[4], Daniella Helena Hock[5], Radiance Lim[2], Rhea Nadkarni[2], Vinh Thang Huynh[3,6], Daryl Lim[7], Wei Leong Chew[7], Franklin L. Zhong[8,9], David Arthur Stroud[5], Sebastian Schafer[2,10], Vinay Tergaonkar[3,11], Ashley L. St John[4,12,13], Owen J.L. Rackham[2], Lena Ho[1,2,3,*]

This PDF file includes:

Supplementary Figs. 1 to 10

Supplementary Table 1. Table containing the sequences of the primers used.

Supplementary Table 2. Table containing the sequences of the primers and probes used to quantify DENV and ZIKV genome.

Supplemental References

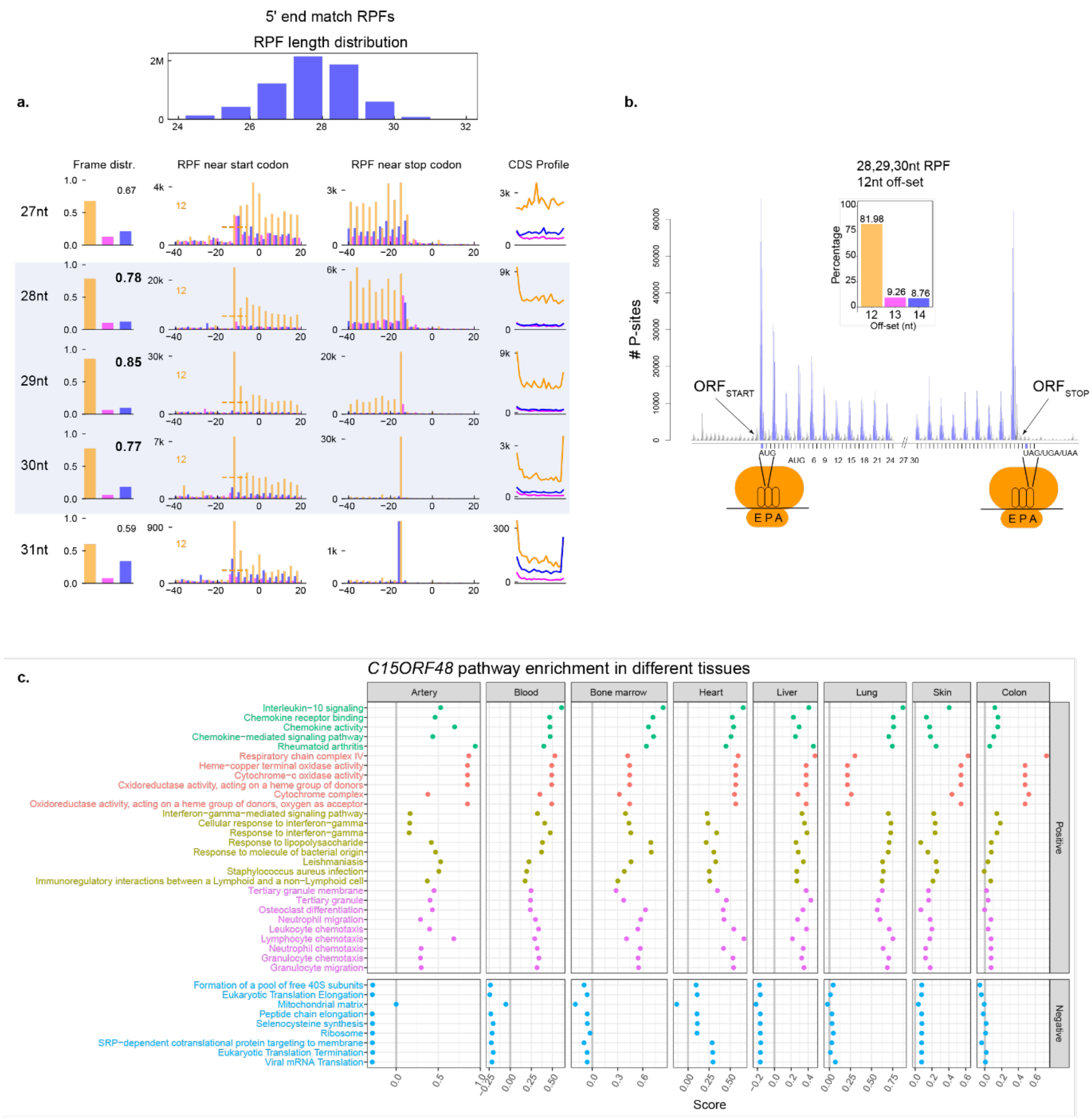

Supplementary Fig. 1. Proteo-genomic screen in human endothelial cells (HAECs) identifies MOCCI as an inflammatory Mito-SEP (*i*Mito-SEP)

a. Distribution of ribosome protected fragment (RPF) lengths in our Ribo-seq dataset. Overall periodicity and offset of each RPF length across annotated open reading frames (ORFs) were further analyzed to determine the optimal values for use in RiboTaper (i.e. 28,29,30 nt read length which showed > 75% in-frame periodicity at an offset of 12 nt).

b. Cumulative distribution of P-site reads at first and last 10 codons of annotated ORFs using RPFs of 28, 29 and 30 nt. Inset shows the percentage of in-frame reads in each frame.

c. Gene module association determination (G-MAD) of MOCCI in different human tissues reveal strong association with inflammation and respiratory chain function. Modules shown have the largest cumulative scores across tissues.

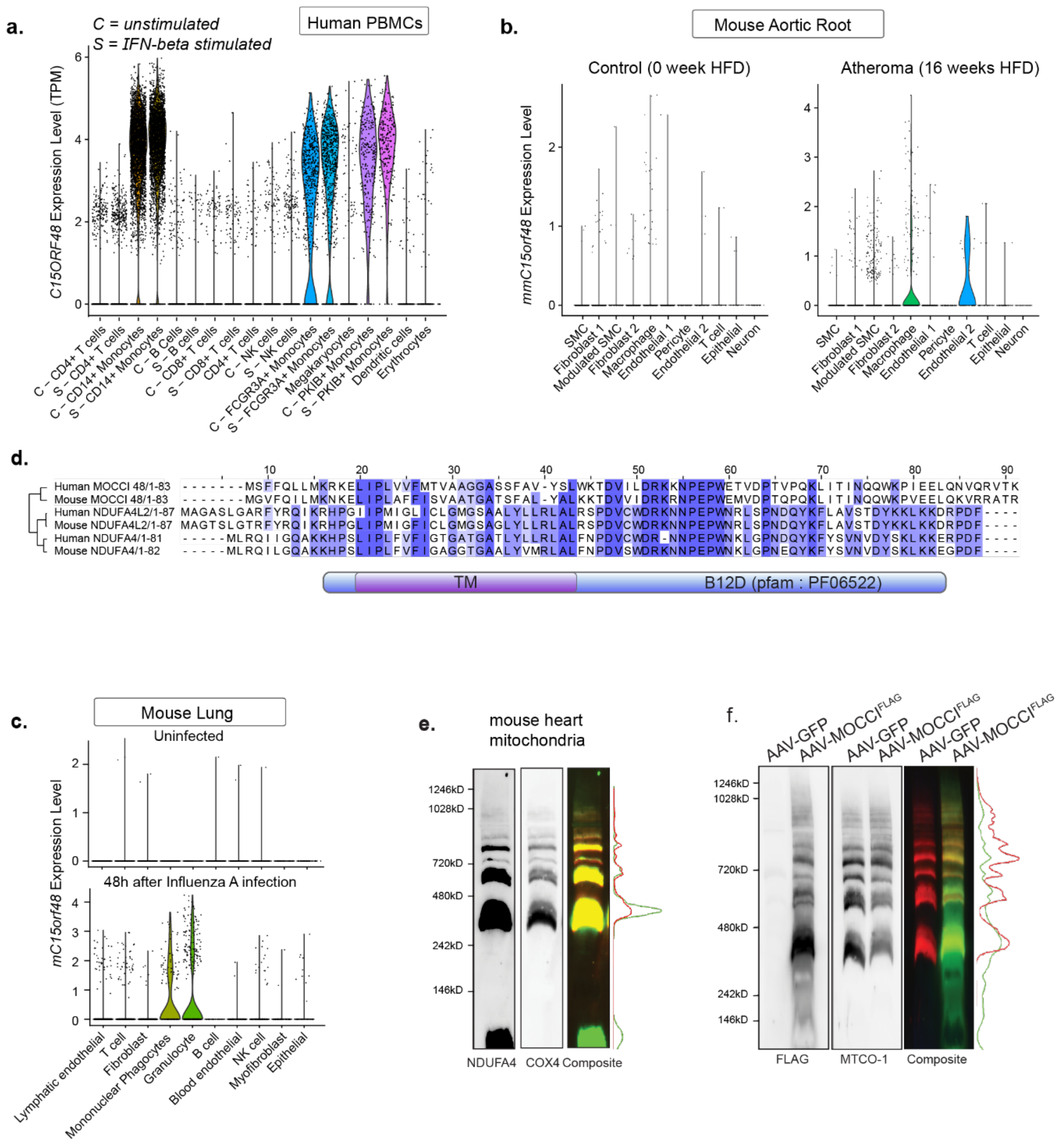

Supplementary Fig. 2 MOCCI is a subunit of Complex IV (Cytochrome C Oxidase)

a. Single cell RNA-seq data of untreated and interferon-treated human peripheral blood mononuclear cells (PBMCs) showing expression levels of *C15ORF48* in both unstimulated and stimulated monocytes. Data set used: GSE96583[1].

b. Single cell RNA-seq data of cells isolated from an *ApoE* knockout high-fat diet (HFD) induced murine atherosclerotic model showing expression of *mC15ORF48* upregulated in macrophages, endothelial cells and modulated smooth muscle cells (SMC) in the atheroma, a region of chronic inflammation. Data set used: GSE131776[2]

c. Single cell RNA-seq data of cells isolated from murine lung following infection by seasonal H1N1 influenza virus showing expression of *mC15ORF48* upregulated in leukocytes as well as lymphatic endothelial cells following acute inflammation. Data set used: GSE107947[3]

d. Alignment of human and mouse NDUFA4, NDUFA4L2 and MOCCI peptides.

e. BN-PAGE of NDUFA4 and COX4 in mouse heart mitochondria. , *n*=4 biological replicates

f. BN-PAGE of FLAG and MTCO-1 in mouse heart mitochondria. , *n*=3 biological replicates

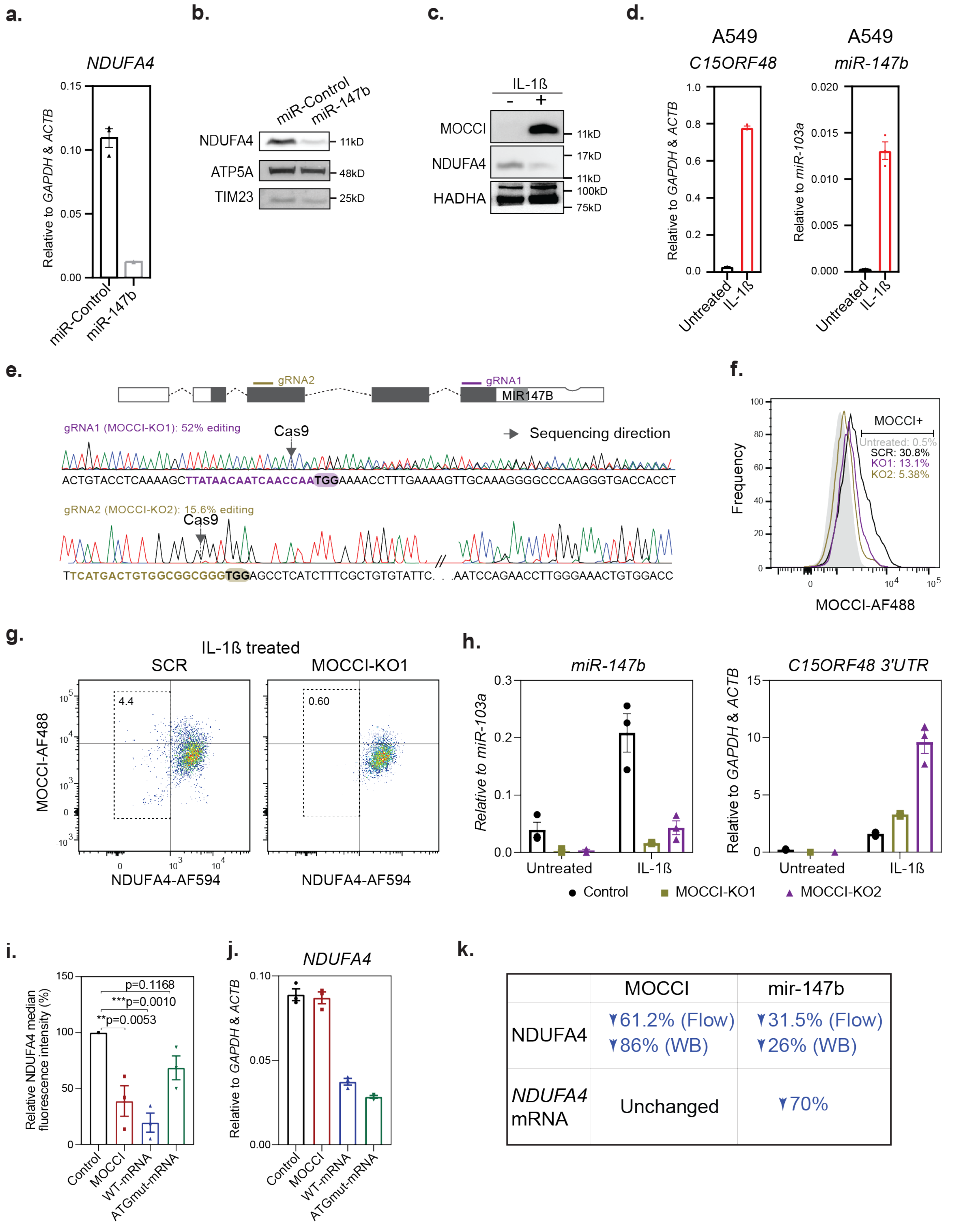

Supplementary Fig. 3 MOCCI and *miR-147b* reduce NDUFA4 expression during inflammation

a. Transfection of *miR-147b* mimic decreased *NDUFA4* transcript levels in HEK293T. Data are presented as mean +/- SEM; *n*= 3 biological replicates per condition

b. Transfection of *miR-147b* mimic decreased NDUFA4 protein levels in HEK293T. Source data are provided as a Source Data file.

c. Immunoblot of MOCCI and NDUFA4 in mitochondria isolated from untreated and IL-1β-treated HAECs. *n*=2 biological replicates. Source data are provided as a Source Data file.

d. *C15ORF48* mRNA and *miR-147b* expression in untreated and IL-1β-treated A549 cells. Data are presented as mean +/- SEM; *n*=3 biological replicates per condition.

e. Schematic of Crispr/Cas9 strategy of generating *MOCCI* ORF knockout in HAECs showing position of 2 gRNAs, and chromatogram of polyclonal edited HAECs after 2 weeks of editing. Percentage of editing was estimated using TIDE[4].
f. Intracellular flow cytometry staining for MOCCI in control (SCR) and gRNA1 (KO1) and gRNA2 (KO2)-edited HAECs following IL-1β stimulated induction as compared to untreated HAECs (grey).
g. Intracellular flow cytometry staining for MOCCI and NDUFA4 in control (SCR) and gRNA1 (KO1)-edited HAECs following IL-1β treatment. Dotted box indicates cells that have undergone NDUFA4 downregulation
h. Normalized miR-147b and *C15ORF48* mRNA levels in control and KO1, KO2 HAECs at basal or following IL-1β stimulus to induce *C15ORF48* expression. Data are presented as mean +/- SEM*;* n= 3 biological replicates per condition.
i. Median fluorescence intensity (MFI) of NDUFA4 collated from 3 biological replicates of data in (Fig 3f), represented as percentage of control. Data are presented as mean +/- SEM P = One-way ANOVA. n= 3 biological replicates per condition
j. Normalized *NDUFA4* mRNA levels in HAECs overexpressing the indicated transgene. Data are presented as mean +/- SEM; n= 3 biological replicates per condition
k. Summary of the effects of MOCCI and miR-147b have on NDUFA4 protein and *NDUFA4* transcript.

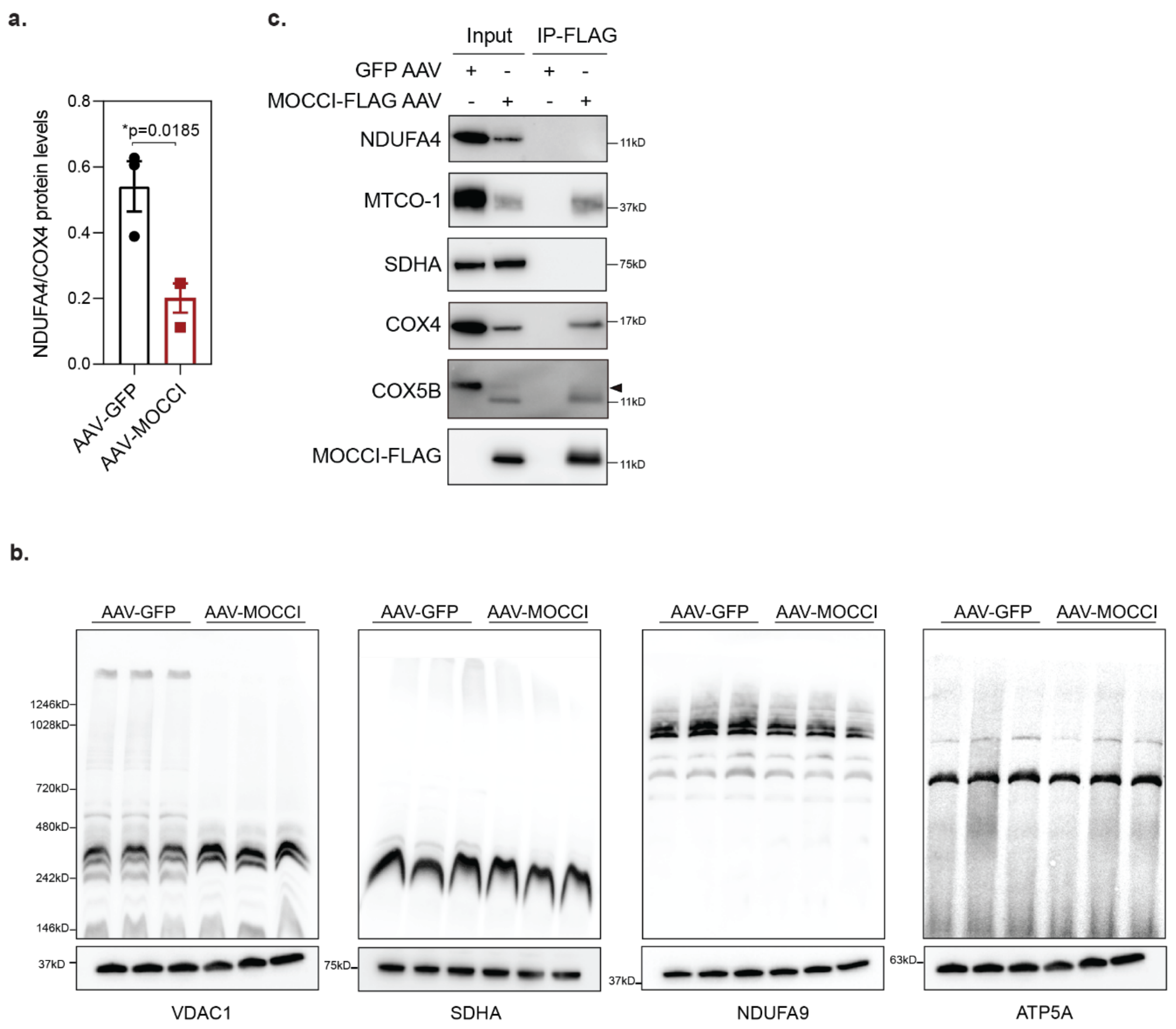

Supplementary Fig. 4 NDUFA4 is exchanged for MOCCI in Complex IV during inflammation

a. Quantitation of NDUFA4 protein levels normalized to COX4 from Fig. 4b. Data are presented as mean +/- SEM; *P* = Two-tailed unpaired Student's t-test; *n* = 3 mice per condition

b. BN-PAGE (top) and SDS-PAGE (bottom) of AAV-MOCCI/GFP mouse heart mitochondria probed for the indicated respiratory chain proteins (SDHA, NDUFA9 and ATP5A). Mitochondrial protein VDAC1 acts as a loading control. Each lane represents mitochondria from one mouse. *n*=3 biological replicates

c. Co-immunoprecipitation (IP) of NDUFA4 and MOCCI in CIV monomer-enriched fractions using sucrose fractionation. Mouse heart mitochondria were immunoprecipitated with anti-FLAG and probed for the proteins as indicated. AAV-GFP served as negative control. *n*=1 biological replicate. Source data are provided as a Source Data file.

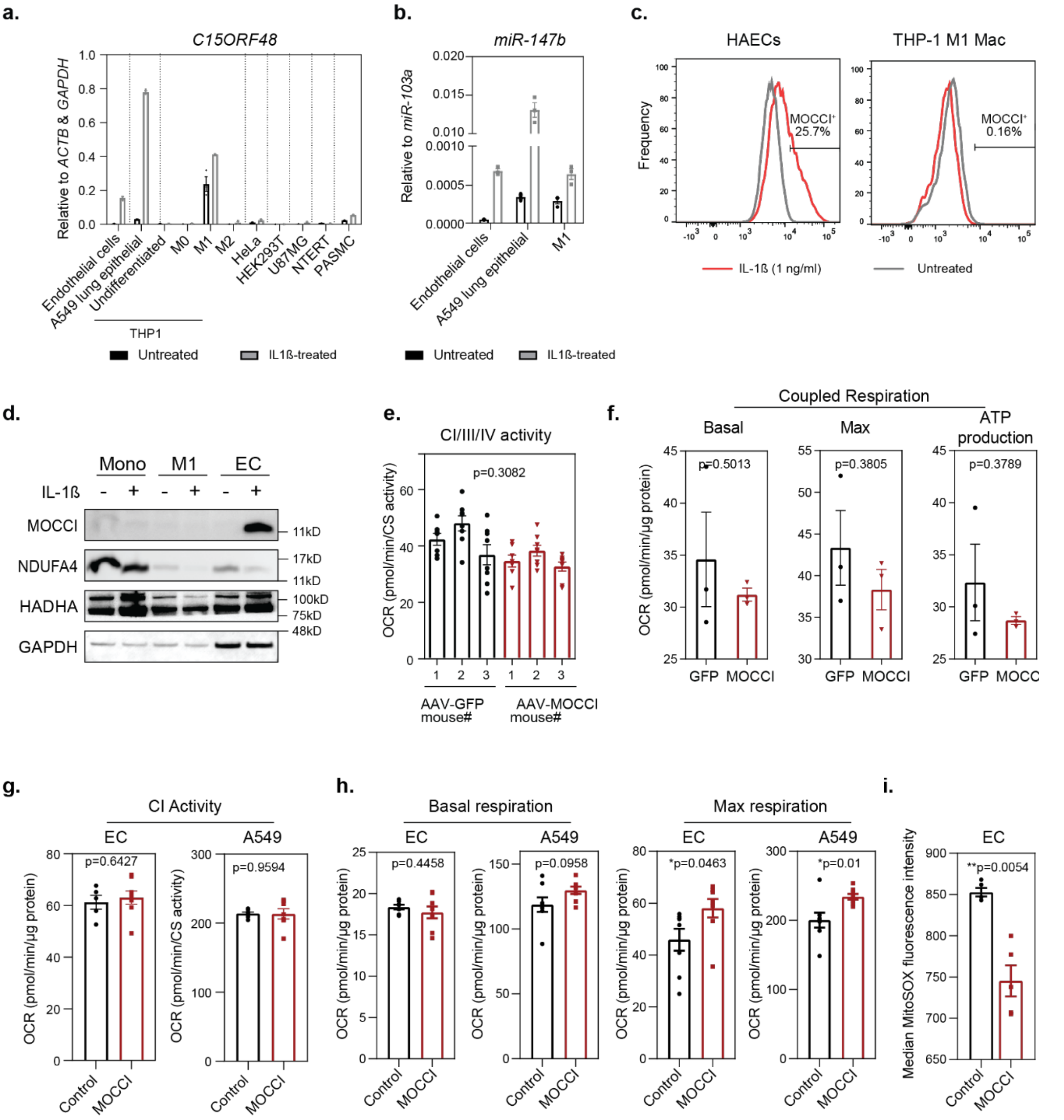

Supplementary Fig. 5 MOCCI reduces CIV activity, membrane potential and ROS production

a. *C15ORF48* mRNA levels by qPCR in different cells at basal or treated with IL-1β. PASMC = Primary pulmonary artery smooth muscle cells. Data are presented as mean +/- SEM; *n*=3 biological replicates per condition.

b. Normalized *miR-147b* levels by qPCR in HAECs, A549 lung epithelial and THP1 M1 macrophages at basal or treated with IL-1β. Data are presented as mean +/- SEM; *n*=3 biological replicates per condition.

c. Intracellular flow cytometry staining for MOCCI in untreated (grey line) and IL-1β-treated (red line) HAECs and THP-1-derived M1 macrophages. n=3 biological replicates per condition.

d. Immunoblot of MOCCI and NDUFA4 in mitochondria isolated from untreated and IL-1β-treated THP1 monocytes, M1 macrophages and HAECs. Results on ECs were used in Supplementary Fig. 3c. *n*=1 biological replicate. Source data are provided as a Source Data file.

e. Uncoupled electron flow from CI to CIII to CIV using pyruvate/malate as electron donors in AAV-MOCCI and AAV-GFP isolated mouse heart mitochondria. Each column represents one mouse, and each dot represents one technical replicate. Data are presented as mean +/- SEM; *P* = Two-tailed unpaired Student's t-test; *n* = 3 biological replicates with 6 technical replicates each.
f. Coupled basal, max respiration and ATP production in response to pyruvate/malate in AAV-MOCCI and AAV-GFP isolated mouse heart mitochondria. Each dot represents 1 mouse and is the average of 6 technical replicates. Data are presented as mean +/- SEM; *P* = Two-tailed unpaired Student's t-test; *n* = 3 biological replicates with 6 technical replicates each.
g. CI activity of permeabilized MOCCI and control cells as measured by Seahorse. Data are presented as mean +/- SEM; *P* = Two-tailed unpaired Student's t-test; *n*= 6 technical replicates each
h. Basal and maximum respiration measured by Seahorse Mito-Stress™ test on MOCCI and control cells. Each dot represents one technical replicate. Data are presented as mean +/- SEM; *P* = One-way ANOVA. *n*= 3 biological replicates
i. Total mitochondrial ROS levels MOCCI and control cells as measured by flow cytometry of MitoSOX dye reaction. Data are presented as mean +/- SEM; *P* = One-way ANOVA; *n*= 3 biological replicates per condition.

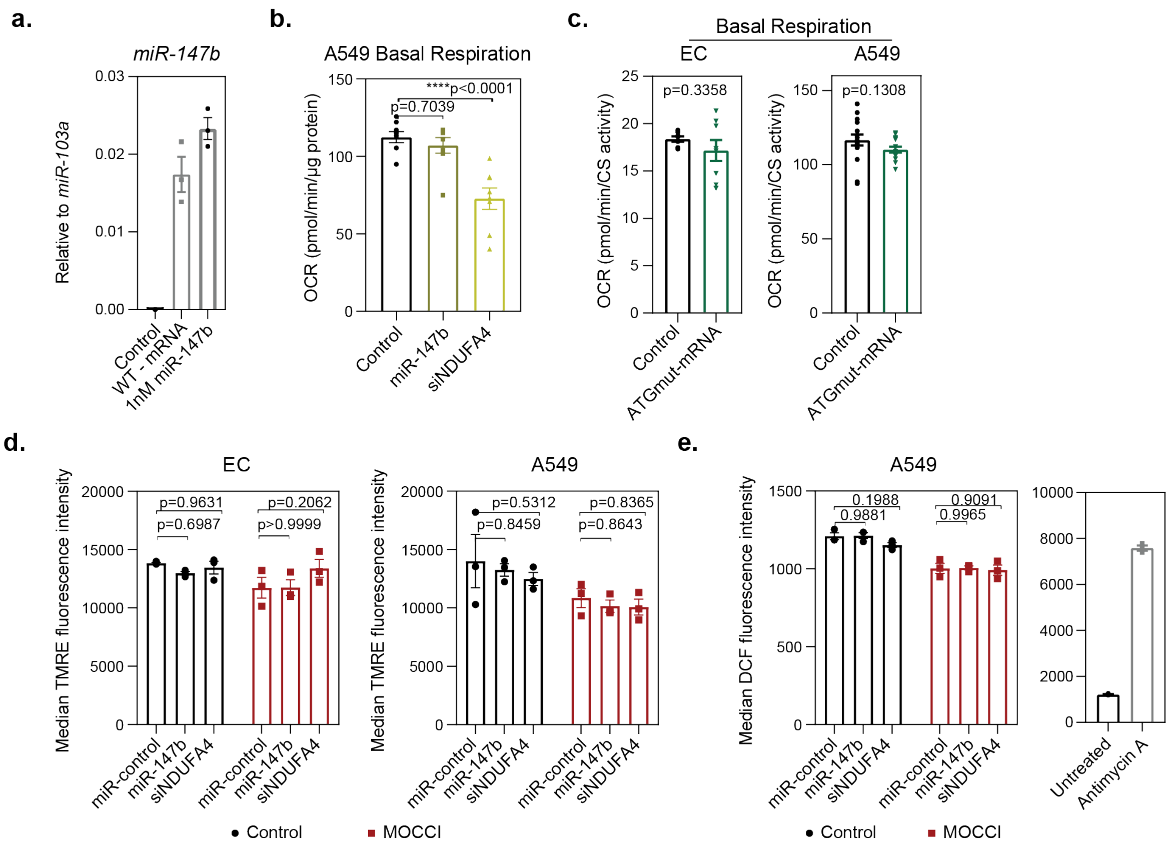

Supplementary Fig. 6 ***miR-147b*** **reduces CIV activity but not membrane potential and ROS production**

a. Normalised levels of *miR-147b* in control cells, cells transduced with *WT-mRNA* and cells transfected with 1 nM *miR-147b* mimic. Data are presented as mean +/- SEM; *n*= 3 biological replicates

b. Basal respiration measured by Seahorse Mito-Stress™ test on A549 cells transfected with scrambled miRNA (Control), *miR-147b* mimic and (miR-147b) siRNA against *NDUFA4* (siNDUFA4). Each dot represents one technical replicate. Data are presented as mean +/- SEM; P = One-way ANOVA; *n*= 8 technical replicates.

c. Basal respiration measured by Seahorse Mito-Stress™ test on ATGmut-mRNA and control cells. Each dot represents one technical replicate. Data are presented as mean +/- SEM; *P* = Two-tailed unpaired Student's t-test; *n*= 8 technical replicates for HAECs and 16 technical replicates over 2 repeats for A549

d. Membrane potential of *miR-control*, *miR-147b* mimic or *siNDUFA4* transfected MOCCI and control cells as measured by flow cytometry of TMRE dye incorporation. Data are presented as mean +/- SEM; *P* = Two-way ANOVA; *n*= 3 biological replicates per condition.

e. (Left) Total cellular ROS levels of miR-control, miR-147b mimic or siNDUFA4 transfected MOCCI and control cells as measured by flow cytometry of DCF dye reaction. (Right) DCF fluorescence intensity of control cells before and after Antimycin A treatment as a positive control. Data are presented as mean +/- SEM; *P* = Two-way ANOVA; *n*= 3 biological replicates per condition.

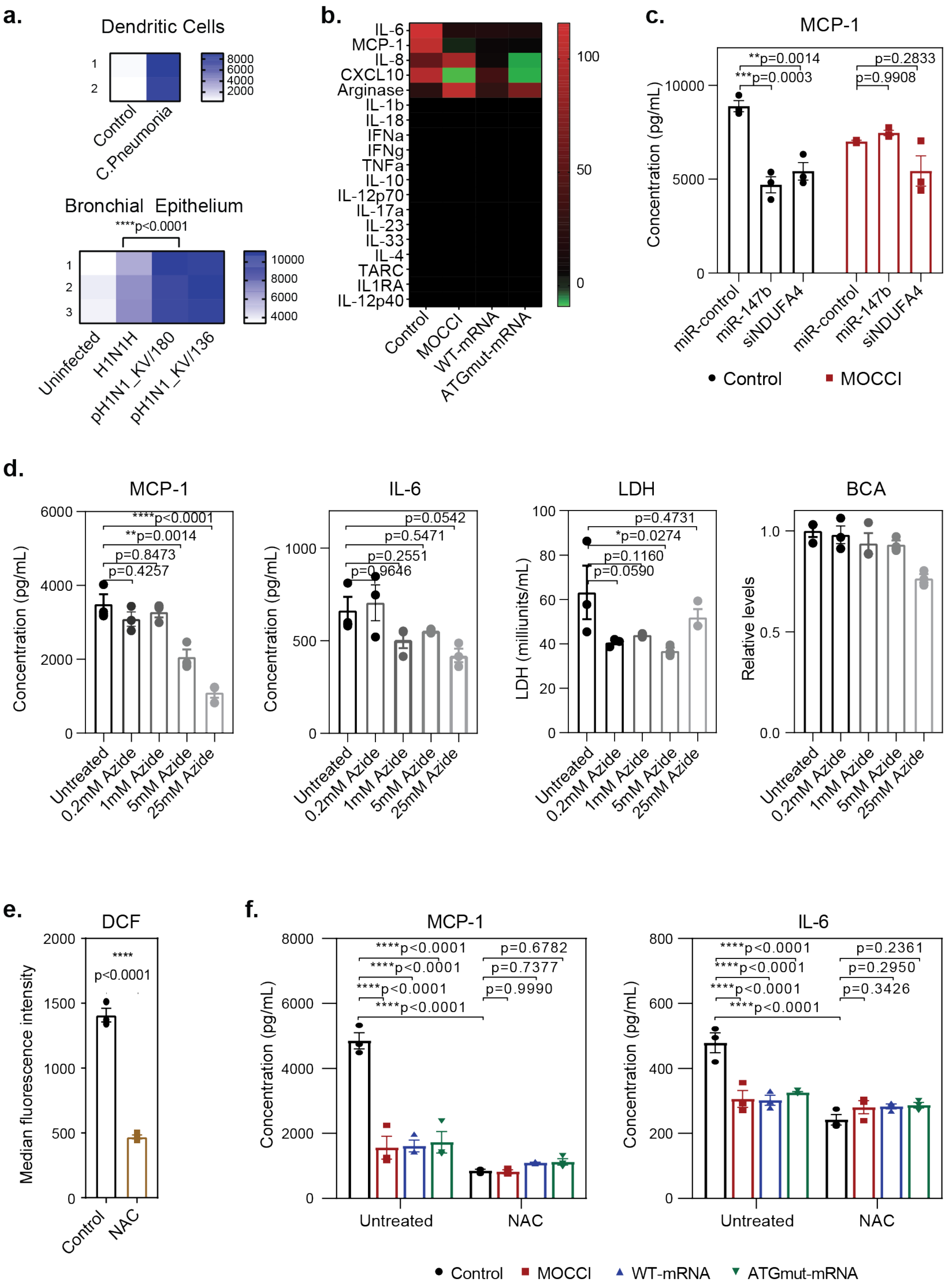

Supplementary Fig. 7 MOCCI and miR-147b reduce MCP-1 and IL-6 secretion during viral infection

a. Levels of *C15ORF48* in indicated RNA-seq datasets of bacterial and viral infection models. Data are extracted from GSE128065 (dendritic cells) and GSE484666 (bronchial epithelium). P = One-way ANOVA

b. Heatmap of the levels of cytokines secreted by HAECs with indicated transgenes following DENV/ZIKV infection, as detected by LEGENDplex cytokine bead array.

c. Levels of MCP-1 (CCL2) secreted by MOCCI or control HAECs transfected with *miR-control*, *miR-147b* mimic or *siNDUFA4* following ZIKV infection. Data are presented as mean +/- SEM; *P* = Two-way ANOVA; *n*= 3 biological replicates per conditions.
d. Levels of MCP-1 (Left panel) or IL-6 (centre left panel) secreted by HAECs treated with indicated concentration of potassium azide. Levels of cytokines were normalized to BCA levels (right panel). (Centre right panel) Lactate dehydrogenase (LDH) levels to show minimal cell death in this assay. Data are presented as mean +/- SEM; *P* = Two-way ANOVA; *n*= 3 biological replicates per conditions.
e. Total cellular ROS levels of HAECs treated with ROS scavenger NAC as measured by flow cytometry of DCF dye reaction. Data are presented as mean +/- SEM; *P* = Two-tailed unpaired Student's t-test; *n*= 3 biological replicates per condition.
f. Levels of MCP-1 (left) and IL-6 (right) secreted by HAECs with indicated transgenes with and without NAC treatment. Data are presented as mean +/- SEM; *P* = Two-way ANOVA; *n*= 3 biological replicates per conditions.

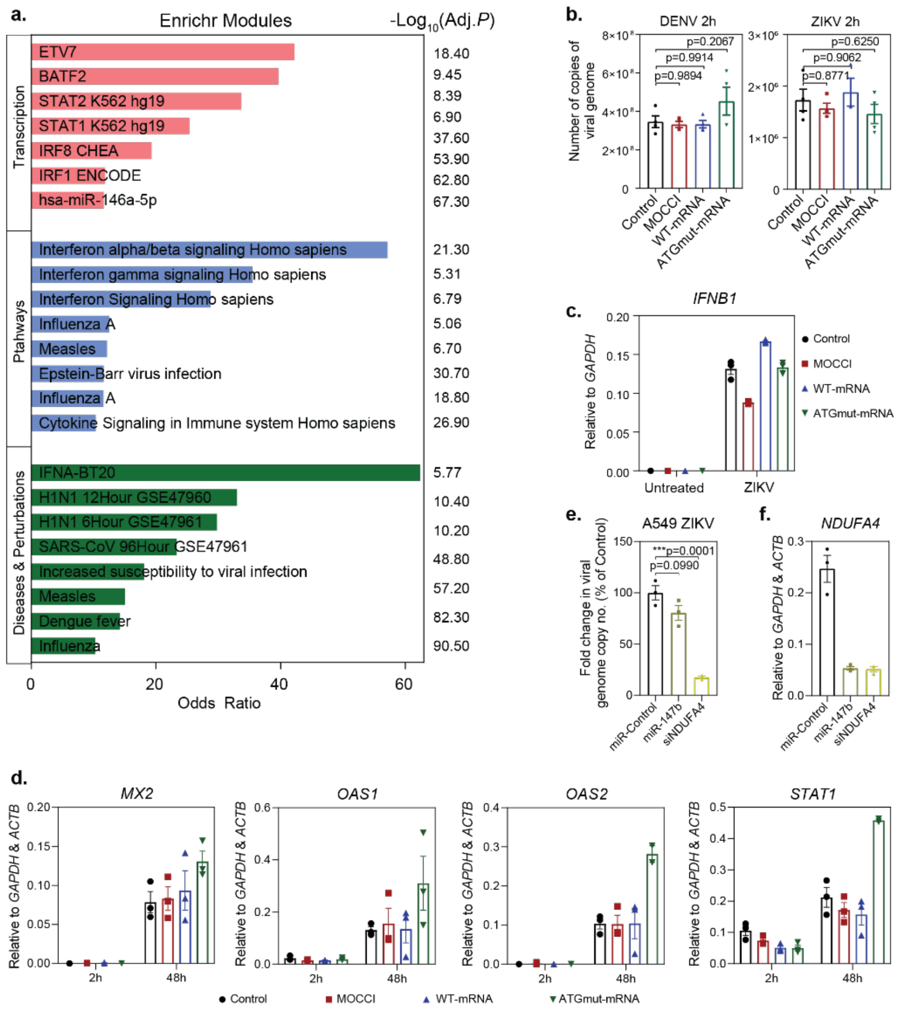

Supplementary Fig. 8 MOCCI and miR-147b co-ordinate to optimize host protection

a. Description of the genes in a. vis-a-vis their transcriptional regulation, pathway membership and involvement in diseases and perturbations as revealed by the Enrichr tool (Ma'ayan lab)[5,6]. Significance levels for the corresponding odds ratio of significant enrichment are reported.

b. HAECs with the indicated transgenes were infected with DENV and ZIKV (MOI = 1.0). 2 hours later, virus copy copy was measured by qPCR-mediated viral genome quantification as a measure of viral entry. Data are presented as mean +/- SEM; P = One-way ANOVA; n = 3 biological replicates per condition.

c. Normalized *IFNB1* mRNA levels in uninfected or ZIKV-infected HAECs overexpressing the indicated transgene. Data are presented as mean +/- SEM; n = 3 technical replicates per condition.

d. Normalized mRNA levels of interferon response genes in 2 h or 48 h ZIKV-infected HAECs overexpressing the indicated transgene. Data are presented as mean +/- SEM; n = 3 biological replicates per condition.

e. A549 transfected with *miR-control*, *miR-147b* mimic or *siNDUFA4* were infected with ZIKV (MOI = 0.1). 48 hours later, viral replication was measured by qPCR-mediated viral genome quantification, compared to a baseline obtained at 2 hours post infection. Values are expressed as percentage of the control of each biological replicate. Data are presented as mean +/- SEM; *P* = One-way ANOVA; *n*= 3 biological replicates per condition.

f. Normalized *NDUFA4* mRNA levels in A549 cells transfected with *miR-control*, *miR-147b* mimic or *siNDUFA4*. Data are presented as mean +/- SEM; n = 3 biological replicates per condition.

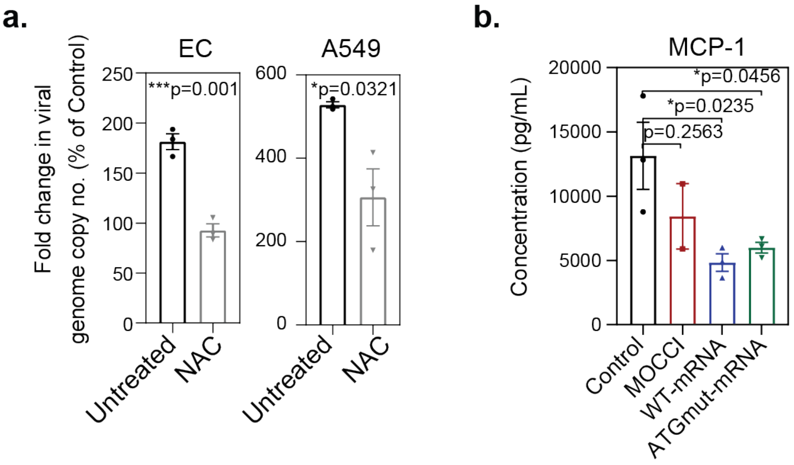

Supplementary Fig. 9 miR-147b works through RIG-I/MDA5 pathway to modulate interferon response

a. HAECs or A549 treated with 1 mM NAC were infected with ZIKV (MOI =1 and 0.1 respectively). 48 hours later, viral replication was measured by qPCR-mediated viral genome quantification, compared to a baseline obtained at 2 hours post infection. Values are expressed as percentage of the control of each biological replicate. Data are presented as mean +/- SEM; *P* = Two-tailed unpaired Student's t-test; *n*= 3 biological replicates per condition.
b. Levels of MCP-1 secreted by A549 with indicated transgenes after transfection with RIG-I agonist. Data are presented as mean +/- SEM; *P* = Two-way ANOVA; *n*= 3 per conditions.

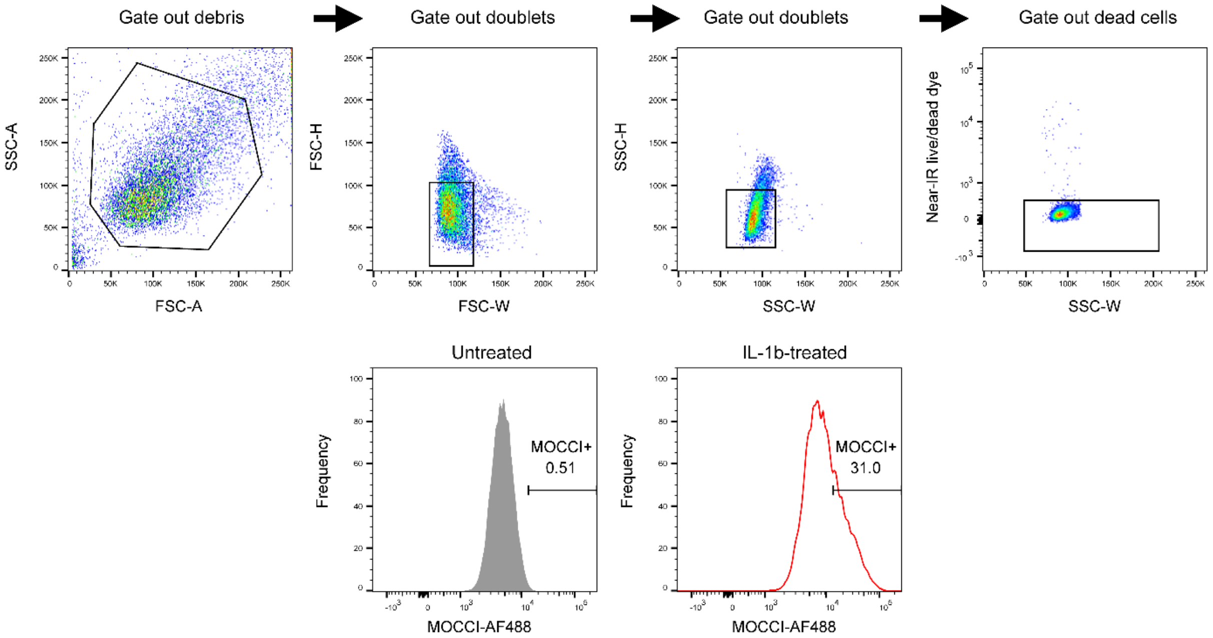

Supplementary Fig. 10 Gating strategy for flow cytometry. Debris, doublets and dead cells were excluded before the cells were analyzed for the genes of interest. This gating strategy was used for Figures 1b, 3a, 3d-f, and Supplementary Figures 3f, 3g and 5c.

Supplementary Table 1. Table containing the sequences of the primers used.

Gene name	Sequence of forward primer	Sequence of reverse primer
ACTB	*TCCCTGGAGAAGAGCTACGA*	*AGCACTGTGTTGGCGTACAG*
GAPDH	*AATCCCATCACCATCTTCCA*	*TGGACTCCACGACGTACTCA*
C15ORF48-ORF	*TGGAGCCTCATCTTTCGCTGTG*	*GCTCGTCATTTGGTCACCCTTT*
C15ORF48 5’UTR	*TGGCAATTCTTCGCTGAAGTC*	*ATCAAGGATCACATCGGTTTTCCA*
C15ORF48 3’UTR	*CGAGCCCTCGCCTCTTTCTTCT*	*GCATTTCCGCACACTGGTGTCC*
IFNB1	*CTTGGATTCCTACAAAGAAGCAGC*	*TCCTCCTTCTGGAACTGCTGCA*
NDUFA4	*TCCTTTCCAGTCGGAGACCT*	*GGGGGATCAAGCTCGGATG*
MX2	AACGTGCAGCGAGCTTGTC	*TGGCTGTTGCTGGAAGGAAT*
OAS1	*TCTGCTGGCTGAAAGCAACA*	*CAGTCCTCTTCTGCCTGTGG*
OAS2	*GAGCCAGTTGCAGAAAACCAG*	*GCATTGTCGGCACTTTCCAA*
STAT1	*CGGCTGAATTTCGGCACCT*	*CAGTAACGATGAGAGGACCCT*

Supplementary Table 2. Table containing the sequences of the primers and probes used to quantify DENV and ZIKV genome.

Purpose	Primer name	Sequence of primer
DENV forward primer	C14A	*AATATGCTGAAACGCGAGAGAAACCGCG*
DENV reverse primer	C69B	*CCCATCTCITCAIIATCCCTGCTGTTGG*
DENV probe	VICD2C38B	*AGCATTCCAAGTGAGAATCTCTTTGTCAGCTGT*
ZIKV forward primer	1086	*CCGCTGCCCAACACAAG*
ZIKV reverse primer	1162c	*CCACTAACGTTCTTTTGCAGACAT*
ZIKV probe	1107	*AGCCTACCTTGACAAGCAGTCAGACACTCAA*

References

1. Kang, H. M. *et al.* Multiplexed droplet single-cell RNA-sequencing using natural genetic variation. *Nat. Biotechnol.* **36**, 89–94 (2018).
2. Wirka, R. C. *et al.* Atheroprotective roles of smooth muscle cell phenotypic modulation and the TCF21 disease gene as revealed by single-cell analysis. *Nat. Med.* **25**, 1280–1289 (2019).
3. Steuerman, Y. *et al.* Dissection of Influenza Infection In Vivo by Single-Cell RNA Sequencing. *Cell Syst.* **6**, 679-691.e4 (2018).
4. Brinkman, E. K., Chen, T., Amendola, M. & van Steensel, B. Easy quantitative assessment of genome editing by sequence trace decomposition. *Nucleic Acids Res.* **42**, e168–e168 (2014).
5. Kuleshov, M. V *et al.* Enrichr: a comprehensive gene set enrichment analysis web server 2016 update. *Nucleic Acids Res.* **44**, W90–W97 (2016).
6. Chen, E. Y. *et al.* Enrichr: interactive and collaborative HTML5 gene list enrichment analysis tool. *BMC Bioinformatics* **14**, 128 (2013).
