# Supplementary material for: Coding and non-coding roles of MOCCI (C15ORF48) coordinate to regulate host inflammation and immunity
Source: Nat Commun. 2021 Apr 9;12:2130. doi: 10.1038/s41467-021-22397-5 (PMC8035321; doi:10.1038/s41467-021-22397-5)
Supplement: Supplementary file 3 — Reporting Summary [file 41467_2021_22397_MOESM3_ESM.pdf]

nature research

Corresponding author(s): Lena Ho

Last updated by author(s): Mar 4, 2020

Reporting Summary

Nature Research wishes to improve the reproducibility of the work that we publish. This form provides structure for consistency and transparency in reporting. For further information on Nature Research policies, see our Editorial Policies and the Editorial Policy Checklist.

Statistics

For all statistical analyses, confirm that the following items are present in the figure legend, table legend, main text, or Methods section.

n/a	Confirmed	
☐	☒	The exact sample size (*n*) for each experimental group/condition, given as a discrete number and unit of measurement
☐	☒	A statement on whether measurements were taken from distinct samples or whether the same sample was measured repeatedly
☐	☒	The statistical test(s) used AND whether they are one- or two-sided<br>*Only common tests should be described solely by name; describe more complex techniques in the Methods section.*
☐	☒	A description of all covariates tested
☐	☒	A description of any assumptions or corrections, such as tests of normality and adjustment for multiple comparisons
☐	☒	A full description of the statistical parameters including central tendency (e.g. means) or other basic estimates (e.g. regression coefficient) AND variation (e.g. standard deviation) or associated estimates of uncertainty (e.g. confidence intervals)
☐	☒	For null hypothesis testing, the test statistic (e.g. *F*, *t*, *r*) with confidence intervals, effect sizes, degrees of freedom and *P* value noted<br>*Give P values as exact values whenever suitable.*
☒	☐	For Bayesian analysis, information on the choice of priors and Markov chain Monte Carlo settings
☒	☐	For hierarchical and complex designs, identification of the appropriate level for tests and full reporting of outcomes
☒	☐	Estimates of effect sizes (e.g. Cohen's *d*, Pearson's *r*), indicating how they were calculated

Our web collection on statistics for biologists contains articles on many of the points above.

Software and code

Policy information about availability of computer code

Data collection	FACS data were collected from BD LSRFortessa using BD FACSDiva 8.<br>Mitostress test data were obtained using Seahorse Wave, Agilent.<br>Confocal images were collected using Olympus FV3000.<br>Optical density was recorded with a Tecan Microplate Reader M200 (Tecan).<br>Chemiluminescence and fluorescence blots were captured by a Chemidoc imaging system (Bio-Rad).<br>qPCR data were acquired by 7900 SDS (Applied Biosciences).
Data analysis	FlowJo version 10.6.1 was used to analyse FACS data.<br>GraphPad Prism 8 was used to do statistical analysis and plot graphs.<br>LEGENDplex v 8.0 software was used to analyse FACS data collected from LEGENDplex cytokine array.<br>R 3.5.1 was used to run the R packages.<br>limma R package was used to compute "CAMERA" function for gene signature.<br>Trimmomatic v0.36, Bowtie v1.2.3, STAR v2.6 and Subread v1.63 were used to anlayse fastq files from RNA-seq and Ribo-seq.<br>Enrichr, DAVID v6.8 and Panther v15.0 were used to determine enrichment for GO terms.<br>DESeq2 R package was used to generate the tables of differentially expressed genes.<br>Seurat v3.1 R package was used to analyze single cell RNA sequencing data.<br>RiboTaper was used to identify open reading frames from Ribo-seq data.<br>Tidyverse R package was used to remove peptides that overlap with longer proteins.<br>Weighted Correlation Network Analysis (WGCNA) R package and DynamicTreeCut R package was used for module detection.<br>Mitochondrial targeting motifs were predicted using the online sites of TargetP, Mitofates, TMHMM v2.0 and SignalP (4.1 and 5).<br>Seahorse Wave, Agilent was used to analyse Mitostress test data and mitochondrial flux assays.<br>Clustal Omega was used to align protein sequence.

Jalview was used to cluster the protein alignment.
iTASSER and Maestro (Schrodinger Suites) were used to predict and construct the structure of peptides.
Tide was used to deconvolute the sequence trace from sanger sequencing.
R libraries ggplot2 and pheatmap were used to generate plots for the figures.
Mass spect data were processed using the MaxQuant platform (version 1.6.10.43) and Perseus (version 1.6.10.43).

For manuscripts utilizing custom algorithms or software that are central to the research but not yet described in published literature, software must be made available to editors and reviewers. We strongly encourage code deposition in a community repository (e.g. GitHub). See the Nature Research guidelines for submitting code & software for further information.

Data

Policy information about availability of data

All manuscripts must include a data availability statement. This statement should provide the following information, where applicable:

- Accession codes, unique identifiers, or web links for publicly available datasets
- A list of figures that have associated raw data
- A description of any restrictions on data availability

The RNA-seq data have been deposited in Sequence Read Archive under the BioProject ID PRJNA672723. The code to identify mito-SEPs are uploaded in GitHub [https://github.com/LenaHoLab/Lee-et-al-2021-R-code]. The mass spectrometry proteomics data have been deposited to the ProteomeXchange Consortium via the PRIDE117 partner repository with the dataset identifier PXD024438. Source data are provided with this paper.

The following data sets from GEO database were used (also listed as a table in Methods):
GEO GSE9820
GSE120862
GSE14905
GSE11223
GSE85791
GSE131776
GSE96583
GSE107947
GSE12806
GSE6863
GSE48466
The following data set from European Genomephenome Archive database was used:
EGAS00001002454
The other databases that were used:
Uniprot
Human MitoCarta2.0
Hallmark gene sets of the Molecular Signatures Database (MSgDB)
IMPI database

Field-specific reporting

Please select the one below that is the best fit for your research. If you are not sure, read the appropriate sections before making your selection.

☒ Life sciences ☐ Behavioural & social sciences ☐ Ecological, evolutionary & environmental sciences

For a reference copy of the document with all sections, see nature.com/documents/nr-reporting-summary-flat.pdf

Life sciences study design

All studies must disclose on these points even when the disclosure is negative.

Sample size	The sample size of experiments are disclosed in the figure legend and commonly accepted samples sizes were used. Sample sizes were chosen based on the previous work using similar methods (Jin Zhang 2012 Nature Protocols; George Savidis 2016 Cell Reports; Zhang Shan et al 2020 Nat comms).
Data exclusions	No data was excluded from the study.
Replication	Experiments were replicated for multiple times as indicated in the figure legends and method.
Randomization	No randomization is required as all cell lines were generated at the same time from the same parental line.
Blinding	The experiments were not blinded as none of the evaluation of the experimental results are subjective. Each experiment was designed with proper controls, and samples for comparison were collected and analyzed under the same conditions.

Reporting for specific materials, systems and methods

We require information from authors about some types of materials, experimental systems and methods used in many studies. Here, indicate whether each material, system or method listed is relevant to your study. If you are not sure if a list item applies to your research, read the appropriate section before selecting a response.

Materials & experimental systems

n/a	Involved in the study
	☒ Antibodies
	☒ Eukaryotic cell lines
☒	☐ Palaeontology and archaeology
	☒ Animals and other organisms
☒	☐ Human research participants
☒	☐ Clinical data
☒	☐ Dual use research of concern

Methods

n/a	Involved in the study
☒	☐ ChIP-seq
	☒ Flow cytometry
☒	☐ MRI-based neuroimaging

Antibodies

Antibodies used

For immunofluorescence:
anti-MOCCI (Sigma Aldrich HPA012943)
anti-TOMM20 (Abcam ab56783)
For immunoblotting:
anti-FLAG (Proteintech 20543-I-AP)
anti-MTCO1 (Abcam ab14705)
anti-MOCCI (Novus Biologicals NBP1-98391)
anti-HADHA (ABclonal A13310)
anti-GAPDH (Cell Signaling Technology 2118)
anti-Histone H3 (Cell Signaling Technology 4499)
anti-NDUFA4 (Bioworld Technology BS3883)
anti-TOMM70 (ABclonalA4349)
anti-TIM23 (Proteintech 11123-1-AP)
anti-citrate synthase (Santa Cruz Biotechnology SC-390693)
anti-COX4 (ABclonal A10098)
anti-UQCRSF1 (Abcam ab14746)
anti-ATP5A (Santa Cruz Biotechnology SC-136178)
anti-SDHA (ABclonal A2594)
anti-VDAC1 (ABclonal A0810)
anti-NDUFA9 (ThermoFisher Scientific 459100)
anti-MDA5 (Cell Signaling Technology 5321)
anti-RIG1 (Cell Signaling Technology 4520)
anti-phosphorylated-p65 (Cell Signaling Technology 3031)
anti-COX5B (Abcam ab110263)
anti-UQCRC1 (ABclonal A3339)

For flow cytometry:
anti-MOCCI (Sigma Aldrich HPA012943)
anti-NDUFA4 (Santa Cruz Biotechnology SC-517091)
anti-ICAM1 (Santa Cruz Biotechnology sc-107 PE)
anti-VCAM1 (Miltenyi Biotec 130-104-127)

The following secondary antibodies were used:
Anti-mouse, rabbit or rat IgG conjugated with HRP (Jackson ImmunoResearch 715-035-150; 111-035-003; 712-066-153)
Alexa Fluor 488, 594 and 647 conjugated secondary antibodies (Invitrogen A11001; A21207; A11008; A11005; A21235)

Validation

Commercial antibodies with credible in-house validations for the right species and literature citations were used. All immunoblotting antibodies were validated by the expected product size of the species according to manufacturer's protocols and published data.
As MOCCI is a novel gene, we did further validation of anti-MOCCI (Sigma Aldrich HPA012943) and anti-MOCCI (Novus Biologicals NBP1-98391) by overexpression and knock out assays.
Anti-NDUFA4 and anti-MOCCI were validated for co-immunofluorescence and flow cytometry by their localisation to the mitochondria. Anti-MOCCI was further validated with overexpression and knockout assays. Anti-NDUFA4 was further validated by its downregulation upon knockdown of its transcript.
anti-ICAM1 and anti-VCAM1 were validated for flow cytometry by the manufacturer.
Anti-TOMM20 was validated for co-immunofluorescence by the manufacturer.

Eukaryotic cell lines

Policy information about cell lines

Cell line source(s)	Human Aortic Endothelial cells and pulmonary artery smooth muscle cells were from Promocell.<br>A549, U87MG, HeLa, HEK293T, , BHK-21, THP1 and Aedes albopictus C6/36 mosquito cells were from ATCC.<br>N/TERT-1 was a generous gift from Rheinwald JG.
Authentication	Human aortic endothelial cells were positive for VE-CAD and CD31. They bind to primers specific to human genes.<br>THP1 cells were obtained from ATCC with the information of validation. They were further validated by their upregulation of M1 and M2 markers upon differentiation with the appropriate cytokines.<br>A549, U87MG, HeLa, N/TERT-1, HEK293T and pulmonary artery smooth muscle cells showed their typical morphology. No additional authentication was performed.
Mycoplasma contamination	All lines tested negative for mycoplasma.
Commonly misidentified lines<br>(See ICLAC register)	No commonly misidentified cell lines were used in the study.

Animals and other organisms

Policy information about studies involving animals; ARRIVE guidelines recommended for reporting animal research

Laboratory animals	8-16 weeks-old C57BL/6J mice of both sexes were used.
Wild animals	This study does not involve wild animals.
Field-collected samples	Field-collected samples are not used in this study.
Ethics oversight	All animal protocols were approved by the NUS Institutional Animal Care and Use Committee (IACUC).

Note that full information on the approval of the study protocol must also be provided in the manuscript.

Flow Cytometry

Plots

Confirm that:

- [x] The axis labels state the marker and fluorochrome used (e.g. CD4-FITC).
- [x] The axis scales are clearly visible. Include numbers along axes only for bottom left plot of group (a 'group' is an analysis of identical markers).
- [x] All plots are contour plots with outliers or pseudocolor plots.
- [x] A numerical value for number of cells or percentage (with statistics) is provided.

Methodology

Sample preparation	To stain for NDUFA4 and MOCCI, cells were trypsinsed and pelleted at 500 g for 5 min. They were then fixed in 1X fixative buffer for 30 min and permeabilised in 1X permeabilisation buffer for 7 min while spinning at 700 g (Life Technologies 00-5523-00). The cells were then blocked in 1% BSA/permeabilisation buffer for 30min, before they were incubated in primary antibodies: anti-MOCCI 1:50 (Sigma Aldrich HPA012943), anti-NDUFA4 1:100 (Santa Cruz Biotechnology SC-517091). After 15 min, they were washed with permeabilisation buffer, before they were stained with secondary antibodies at 1:1000 (Alexa Fluor 488, 647, Invitrogen). After 15 min, they were washed again and fixed in 2% PFA. The fluorescence intensity was acquired by LSRFortessa™ (BD Biosciences).<br>To stain for surface proteins, the cells were trypsinised, pelleted and blocked in 1% BSA/PBS. The cells were then incubated with Near-IR Live/Dead dye (Life Technologies L10119) and primary antibodies for 30 min on ice. The antibodies used were anti-ICAM1-PE 1:100 (Santa Cruz Biotechnology sc-107 PE), anti-VCAM1-PE-Vio770 1:100 (Miltenyi Biotec 130-104-127). After washing with 1% BSA/PBS, the cells were fixed in 2% PFA before their fluorescence intensity was acquired by LSRFortessa™ (BD Biosciences).
Instrument	LSRFortessa™ (BD Biosciences)
Software	FlowJo
Cell population abundance	Flow cytometry was used only for analysis and cells were not sorted.

Gating strategy	Debris were gated out using FSC-A and SSC-A. Single cells were then selected for based on their FSC-H vs FSC-W and their SSC-H vs SSC-W. Dead cells were then excluded based on the Live/dead dye. As MOCCI is not expressed in untreated cells, MOCCI+ cells are defined as cells with fluorescence intensity higher than 99.5% of the untreated cells.

☒ Tick this box to confirm that a figure exemplifying the gating strategy is provided in the Supplementary Information.
