# Supplementary material for: Coding and non-coding roles of MOCCI (C15ORF48) coordinate to regulate host inflammation and immunity
Source: Nat Commun. 2021 Apr 9;12:2130. doi: 10.1038/s41467-021-22397-5 (PMC8035321; doi:10.1038/s41467-021-22397-5)
Supplement: Supplementary file 4 — Description of Additional Supplementary Files [file 41467_2021_22397_MOESM4_ESM.pdf]

Description of Additional Supplementary Files

File Name: Supplementary Data 1
Description: List of *i*-Mito-SEP candidates identified by RiboTaper from Ribo-seq data that are differentially regulated as detected by RNA-seq

File Name: Supplementary Data 2
Description: Table of proteins identified by mass spectrometric analysis of isolated heart mitochondria from AAV-mMOCCI and AAV-GFP mice
